# Peri-Implant Oral Squamous Cell Carcinoma (OSCC): Clinicopathological Features and Staging Issues

**DOI:** 10.3390/cancers17132149

**Published:** 2025-06-26

**Authors:** Luisa Limongelli, Fabio Dell’Olio, Antonio D’Amati, Eliano Cascardi, Marta Forte, Rosaria Arianna Siciliani, Alfonso Manfuso, Eugenio Maiorano, Gianfranco Favia, Chiara Copelli, Saverio Capodiferro

**Affiliations:** 1 Odontostomatology Unit, Interdisciplinary Department of Medicine, Aldo Moro University of Bari, 70125 Bari, Italy; luisa.limongelli@uniba.it (L.L.); marta.forte@uniba.it (M.F.); r.siciliani2@studenti.uniba.it (R.A.S.); gianfranco.favia@uniba.it (G.F.); saverio.capodiferro@uniba.it (S.C.); 2 Pathology Unit, Department of Precision and Regenerative Medicine and Ionian Area (DiMePRe-J), University of Bari “Aldo Moro”, 70124 Bari, Italy; antonio.damati@uniba.it (A.D.); eugenio.maiorano@uniba.it (E.M.); 3 Maxillofacial Surgery Unit, Interdisciplinary Department of Medicine, Aldo Moro University of Bari, 70125 Bari, Italy; alfonso.manfuso@policlinic.ba.it (A.M.); chiara.copelli@uniba.it (C.C.)

**Keywords:** oral squamous cell carcinoma, dental implant, peri-implantitis, peri-implant malignancy, surgery, oral pathology, survival, oral potentially malignant diseases

## Abstract

**Background:** Eighty-five percent of peri-implant malignancies are oral squamous cell carcinomas (OSCCs), and most of them are misdiagnosed as peri-implantitis because of their clinical and radiological presentation; few studies have focused on addressing and solving the diagnostic issues related to peri-implant OSCCs. **Objectives:** The study aimed to describe the clinicopathological features of peri-implant OSCCs and to report the staging issues related to the diagnosis of these lesions. **Methods:** This retrospective cohort study included patients who received a diagnosis of and treatment for peri-implant OSCCs at the Unit of Dentistry of the “Aldo Moro” University of Bari (Italy) from 2018 to 2024. By using descriptive statistics, the authors highlighted the diagnostic issues related to the clinical presentation, radiological features, and histology of peri-implant OSCCs. **Results:** A total of 13 women and 8 men with a mean age of 70.6 ± 11.7 years met the inclusion criteria; the medical history of the participants showed potentially malignant disorders (OPMDs) in 52.4% of patients, whereas 14.3% had already developed an OSCC. The patients showed 24 peri-implant OSCCs; the clinical presentation was leuko-erythroplakia-like (41.7%) or erythroplakia-like (58.3%), thus simulating peri-implantitis; in addition, 52.0% of dental implants involved had a probing pocket depth ≥ 10 mm, further mimicking peri-implantitis. Panoramic radiograms and cone beam computed tomography were of little use in studying bundle bone–implant interfaces; in particular, the tomography showed circumferential bone resorption only in peri-implantitis-like OSCCs. In total, 91.6% of histological examinations of OSCCs showed peri-implantitis-like inflammation; early-stage lesions (pTNM I-II) accounted for 33.3%, whereas late-stage lesions (pTNM III-IV) accounted for 66.7%; lymph nodal metastases occurred in 25.0% and 62.5%, respectively. The mean follow-up was 3.4 ± 1.0 years; all patients with OPMDs had poorly differentiated tumors and thus showed a worse prognosis than those without OPMDs (mean disease-free survival of 15.5 ± 7.7 months and 44.7 ± 12.1 months, respectively). **Conclusions:** The results of the study showed that peri-implant OSCCs occurred most frequently in patients with OPMDs or previous OSCC; in addition, peri-implant OSCCs required demolition rather than conservative excision, and the prognosis of patients strictly depended on the grade of the cancer. In the authors’ experience, the clinical–radiological presentation simulating peri-implantitis was the feature that concurred most in complicating the diagnosis of those tumors.

## Simple Summary

The most frequent peri-implant malignancies are oral squamous cell carcinomas; most of these cancers are misdiagnosed as peri-implantitis because of their clinical and radiological presentation, thus raising several diagnostic issues. This retrospective cohort study aimed to describe the clinicopathological features of peri-implant oral squamous cell carcinomas and to report the staging issues related to the diagnosis of these lesions; the authors included patients who received a diagnosis of and treatment for peri-implant oral squamous cell carcinomas at the Unit of Dentistry of the “Aldo Moro” University of Bari (Italy) from 2018 to 2024. The results of the study showed that these cancers occurred most frequently in patients with oral potentially malignant disorders or a history of oral squamous cell carcinomas; in addition, peri-implant oral squamous cell carcinomas required demolitive surgery rather than conservative excision, and the prognosis of patients depended strictly on the grade of the cancer. In the authors’ experience, the clinical–radiological presentation simulating peri-implantitis was the feature that concurred most in complicating the diagnosis of those tumors. Such findings will be useful for reducing the chances of misdiagnosis of peri-implant oral squamous cell carcinomas.

## 1. Introduction

Oral squamous cell carcinoma (OSCC) is a malignant epithelial neoplasm that counts for more than 90% of all oral malignancies and is the cancer that ranks sixth for worldwide prevalence [1,2,3,4,5]. The other subtypes of oral cancer show a lower incidence than OSCC and include fusiform, papillary, verrucous, mucoepidermoid, adenomatous, acantholytic, and cuniculatum carcinomas [5,6,7]. The risk factors for OSCC include smoking habits, alcohol consumption, nutritional deficiencies, betel quid chewing, ionizing radiation, immunosuppressants, and oral potentially malignant disorders (OPMDs) [1,2,8]. The peak incidence of OSCC occurs in men over 60 years; however, such malignancy also showed a high incidence in patients under 40 years and without risk factors in medical history [1]. The 5-year relative survival rate of stage I-II OSCC is 86%; the rate is 69% in patients with stage III OSCC and 40% in those with stage IV [9]. Oral rehabilitation by osseointegrated dental implants is a predictable and successful technique; thus, it is one of the best options for edentulism [1,10,11]. Peri-implantitis (PI) is a frequent complication of implantology and occurs in up to 20% of osseointegrated dental implants [1,4,10,12,13]. PI is a plaque-dependent chronic inflammatory pathology that affects the peri-implant mucosa and causes the resorption of the supporting bone and the formation of bone pockets with progressive deep extension, according to the definition by the World Workshop on the Classification of Periodontal and Peri-implant Diseases and Conditions [13]. While there is general agreement on the diagnostic criteria for PI [13,14], interpretation can still vary among clinicians. Moreover, although no standardized treatment protocol exists, numerous therapeutic approaches have been proposed and are widely used. It should also be clarified that histological examination is not a routine part of PI treatment protocols but is indeed advisable when clinical suspicion arises. The clinical presentation of PI shows edema, swelling, erythema, hypertrophy, suppuration, pocket formation, bone loss, ulcers, and even granular hyperplastic changes of the soft tissues; therefore, a differential diagnosis with malignant lesions is necessary in these cases [1,3,4,12,15,16,17]. In addition, PI and OSCC share a smoking habit as a conventional risk factor [8]. Several authors reported OSCC as the most frequent peri-implant malignancy (85%), followed by osteosarcoma, plasmacytoma, and lymphoma [4,15,18]. Metastases occur in 9% of peri-implant malignancies and develop mainly from breast and lung cancers [4,15,18]. Several authors hypothesized that dental implants could facilitate tumor invasion in soft tissues and peri-implant bone [16]. However, the literature lacks a dedicated staging system for peri-implant OSCC; a few studies have addressed the importance of adapting the staging system and the type of resection based on the distance of the malignancies from the dental implants [16]. The current retrospective cohort study aims to describe the clinicopathological features and staging issues of peri-implant OSCC based on the diagnosis, therapeutic management, and prognosis of the cases referred to the Unit of Dentistry of the “Aldo Moro” University of Bari (Italy) from 2018 to 2024.

## 2. Materials and Methods

The authors designed a retrospective cohort study, which complied with the Declaration of Helsinki and received the approval of the local Ethical Committee (study 4574; code 1442/CE). Before joining the study, all participants provided written informed consent for diagnostic and therapeutic procedures for research purposes.

### 2.1. Inclusion Criteria and Participants

The authors included adult patients who showed any OSCC with clinical involvement of one or more dental implants and who received histological diagnosis and treatment at the Unit of Dentistry of the “Aldo Moro” University of Bari, Italy, from January 2018 to January 2024. A total of 13 women and 8 men (Table 1; F/M ratio = 1.6) with a mean age of 70.6 ± 11.7 years met the inclusion criteria and joined the study (participation rate: 100%; *n* = 21).

### 2.2. Clinical Workflow

#### 2.2.1. Diagnostic Phase and Clinical Staging

All patients underwent the World Health Organization’s eight-step clinical examination; then, the authors took photographic check-ups of the peri-implant lesions before and after applying toluidine blue and Lugol’s solution [19,20]. The authors completed the clinical examination of the lesions by assessing the signs of PI according to the Classification of Periodontal and Peri-implant Diseases and Conditions published in 2018 [13]. The periodontal chart recorded bleeding, suppuration, a probing pocket depth (PPD) over 6 mm, crestal bone loss not addressable with physiologic bone remodeling, and a bone level 3 mm below the most coronal portion of the intra-osseous part of the implant [13]. Each patient underwent periapical radiographs, a panoramic radiogram, and high-definition multi-slice spiral computed tomography (CT) with three-dimensional reconstruction to assess the conditions of the peri-implant bone. The authors gathered the medical history of all patients by paying attention to risk factors for oral cancer, previous diagnoses of OPMDs or OSCCs, and malignancies in other areas. The authors also collected information on the dental implants associated with the lesions, such as the reasons for the tooth loss, the delay between the placement and the occurrence of the peri-implant lesion, and previous diagnosis and treatment for PI. As the first intervention, all patients received cleaning of the implant fixtures with plastic scaling instruments and ultrasonic scalers with a non-metallic tip [21]. The authors also performed submucosal treatment by diode laser and antimicrobial application in peri-implant pockets (metronidazole 40% gel) [21]. After two weeks, all patients with persisting lesions underwent a cytologic examination of the peri-implant sulcus by brushing to prepare five to six monolayer slides for each lesion [19,22]; then, patients with a cytologic diagnosis of cells with atypias suggestive of carcinoma received a single or multifocal incisional biopsy. The patients who showed evidence of malignancy underwent magnetic resonance imaging (MRI) to measure the extension of the lesions, assess the infiltration of cervical lymph nodes, and complete the cTNM staging according to the American Joint Commission on Cancer (AJCC), 8th Edition TNM guidelines [23].

#### 2.2.2. Surgical Phase and Pathological Staging

The patients underwent the surgical excision of the lesion under general anesthesia and clinical–radiological follow-up of the locoregional lymph nodes for early detection of metastases. The surgical excision was cold-blade and extended to healthy wide margins for 1 cm; after the excision of the soft tissue lesion, the surgeon sampled the lateral and inner deep margins [19]. Then, the authors performed an en bloc bone resection to remove the implant fixtures and the surrounding bone for histopathological diagnosis of the cancers with clinical evidence of bone invasion. The pathologists performed the intraoperative histological examination on frozen sections of the whole sample to confirm the diagnosis of malignancy; in addition, the assessment of the margins aimed to check for tumor infiltration [19]. Once the pathologists established the margins as 0.5–1 cm free of disease, the surgeon transitioned to the reconstructive step with local or distant vascularized flaps. The maxillofacial surgeons excised the lymph nodes in patients with clinical–radiological signs of metastases and in those who showed peri-implant OSCCs invading the bone; in addition, the patients who underwent lymph node excision received adjuvant radiotherapy or chemotherapy after assessment by the institutional tumor board. Those with distant metastases received chemotherapy.

#### 2.2.3. Follow-Up

The authors fixed the samples for the standard histopathological examination in 10% neutral buffered formalin. The pathologists decalcified the bone samples in formic acid 5% in distilled water for 24 h; then, they embedded the bone samples in paraffin, sectioned them, and stained them with hematoxylin–eosin for traditional microscopy [21]. All patients underwent a follow-up protocol based on intraoral clinical examination, ultrasound assessment of lymph nodes, oncologic visits, and instrumental monitoring of distant metastases. The authors scheduled the follow-up once per month during the first postoperative year, every two months during the second, every three months during the third, and every six months up to the fifth; then, the patients underwent clinical and ultrasound follow-up once a year [19].

### 2.3. Statistical Methods

The authors performed a descriptive statistical analysis using the software Stata 16 (StataCorp, College Station, TX, USA, 2019); in particular, the authors reported continuous quantitative variables using means and standard deviations, while qualitative variables used absolute and relative frequencies. The authors analyzed demographic, preoperative, histological, and postoperative outcomes. The demographic outcomes involved the incidence of peri-implant OSCC, mean age, and sex. The preoperative outcomes counted the clinical stage (cTNM), the presentation, the localization, the elapsed time between the implant placement and diagnosis of a tumor, previous oral cancers and malignancies in other areas, OPMDs, risk factors for oral cancer, the efficacy of the panoramic radiogram and high-definition multi-slice spiral CT in the visualization of the cancers, the number of implants involved in the malignancy, and the type of prostheses. The histological outcomes listed the pathological staging (pTNM), the grading of the cancers, the occurrence and the extent of bone invasion, and the prevalence of PI-like inflammation; moreover, the authors distinguished the pathological stage assessed in the tissues sampled by incisional biopsy from the stage assessed after the excision with en bloc resection. The postoperative outcomes were the disease-free survival (DFS), the overall survival (OS), and the mean follow-up time.

## 3. Results

### 3.1. Demographic and Preoperative Outcomes

The mean incidence of peri-implant OSCCs was 4.2 ± 1.9 cases per year, which ranged from a minimum of 1 to a maximum of 7. The treated peri-implant OSCCs were 24 in number because three patients showed 2 synchronous lesions of this kind. In addition, three other patients showed multiple synchronous OSCCs in regions other than peri-implant regions (14.3%).

The clinical presentation of the peri-implant OSCCs was erythroplakia-like in 14 cases (58.3%); in such cases, the peri-implant mucosa showed bleeding and slight swelling, and simulated PI. The remaining 10 peri-implant OSCCs occurred as exophytic mixed red and white lesions (41.7%); the authors defined such clinical presentations as micronodular leukoplakia-like. According to the cTNM staging system, the patients showed 9 stage I peri-implant OSCCs (37.5%), 12 stage II OSCCs (50.0%), 1 stage III OSCC (4.2%), and 2 stage IVa OSCCs (8.3%). Peri-implant OSCCs occurred in the mandible more often than in the maxillae (19 vs. 5, respectively; 79.2% vs. 20.8%). The medical history of 17 patients showed risk factors for peri-implant OSCC (81.0%; Table 2); 3 had a previous OSCC in a site other than the site of peri-implant cancer (14.3%), 4 had proliferative verrucous leukoplakia (PVL) (19.0%), 5 had oral lichen planus (OLP) (23.9%), and 2 had lichenoid dysplasia (9.5%). Nine patients reported smoking habits in their medical history (42.9%); all patients with previous OSCCs were former smokers, whereas the remaining six belonged to the groups with and without OPMDs. All data regarding the OPMDs were derived from histological examination of biopsies that the patients underwent before developing the peri-implant OSCCs.

**Figure 1:**
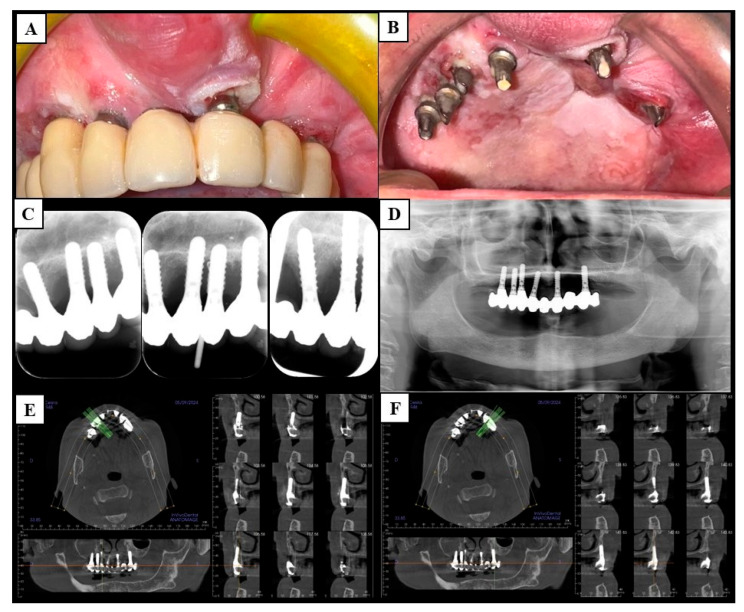
Clinical case 1. The figure shows a patient with multiple synchronous maxillary peri-implant squamous cell carcinomas and focuses on the lesion in region 2.1. (**A**,**B**): Clinical presentation with and without the porcelain-fused-to-metal total fixed prosthesis, respectively. From (**C**–**F**): Panoramic radiogram and high-definition multi-slice spiral computed tomography are inadequate for assessing the peri-implant bone invasion, which mimics peri-implantitis; the intraoral radiograms show more details of the peri-implant bone resorption than tomography; however, the neoplastic bone resorption is undistinguishable from peri-implantitis anyway. Figure 3 shows the histological examination of the lesion.

**Figure 2:**
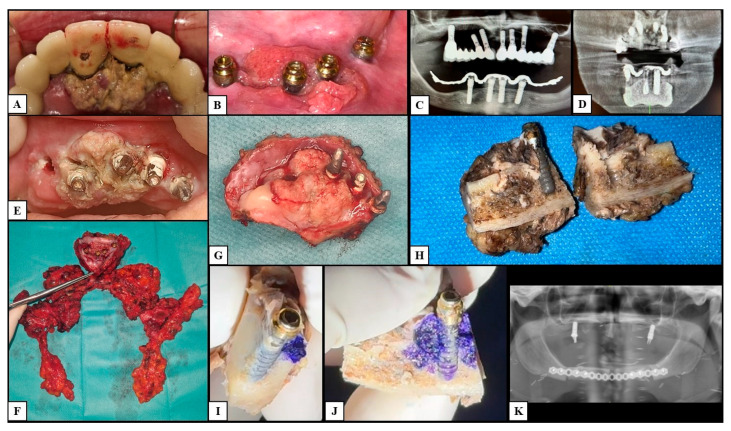
Clinical case 2. This figure shows a patient with multiple synchronous late-stage peri-implant squamous cell carcinomas of the upper and lower jaws. (**A**,**B**): Clinical presentation of the maxillary and mandibular cancers, respectively. (**C**,**D**): Panoramic radiogram and high-definition multi-slice spiral computed tomography of the lesions showing nonspecific peri-implant bone resorption mimicking early stages of peri-implantitis. (**E**): An intraoperative image was taken by removing the upper porcelain-fused-to-metal partial fixed prosthesis. (**F**,**G**): Intraoperative macroscopic specimens with resected mandibular bone and bilateral lymph nodes. From (**H**–**J**): Macroscopic paraffine-embedded specimens with toluidine blue showing the carcinoma and the peri-implant bone invasion. (**K**): Postoperative panoramic radiogram. Figure 4 shows the histological examination of the mandibular lesion.

Panoramic radiograms and high-definition multi-slice spiral computed tomography (CT) were of little use in studying bundle bone–implant interfaces. The panoramic radiograms allowed the detection of the implants in the region of OSCCs, whereas the CTs showed circumferential bone resorption only in PI-like OSCCs. Anyway, the scattering of titanium prevented the study of the bone–implant interface in all cases (Figure 1 and Figure 2). In addition, MRI provided further information to study the bone invasion in small malignancies, in which CT was impaired because of the scattering caused by the dental implants themselves. In total, the dental implants involved were 50 in number (2.1 ± 2.3 per lesion), and 26 of those had a probing pocket depth ≥ 10 mm (52.0%). The peri-implant OSCCs developed after a mean period of 35.5 ± 13.8 months from the dental implant placement; the minimum was 16 months and occurred in a patient with a previous OSCC. A total of 13 patients had porcelain-fused-to-metal (PFM) partial fixed prostheses (61.9%); the remaining patients had overdentures, 5 cemented (23.9%), 2 screwed (9.5%), and 1 removable (4.7%). After removing the prostheses, the authors experienced that tissue-level dental implants allowed better clinical visualization of the lesions than bone-level implants.

### 3.2. Histological and Postoperative Outcomes

**Figure 3:**
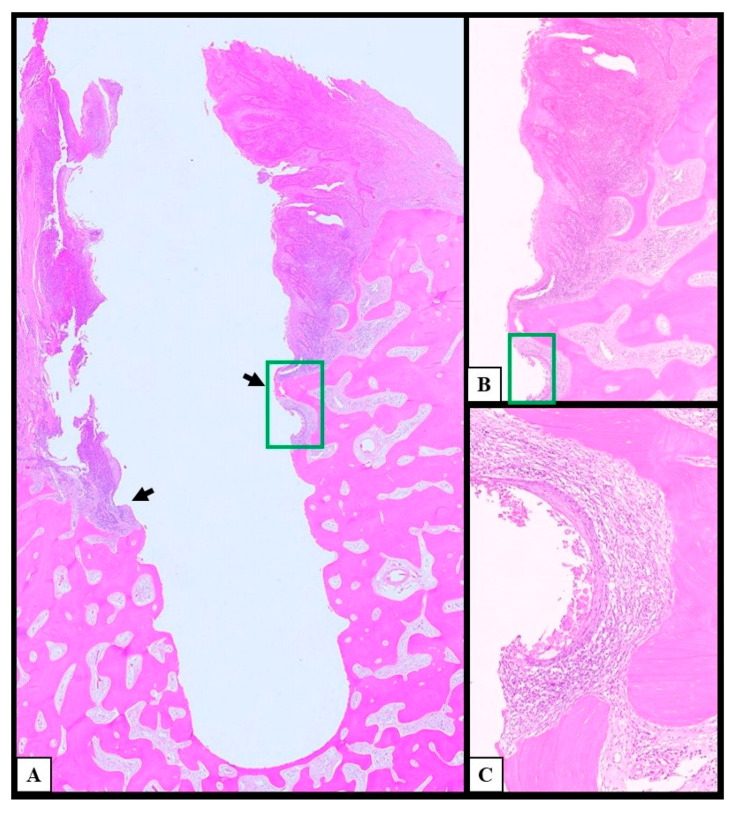
Histological analysis of a late lesion. The figure shows histological images of a late peri-implant squamous cell carcinoma; pictures (**A**–**C**) are different magnifications of the same section. (**A**): In this low-magnification picture of a squamous cell carcinoma, the arrows point to the bone resorption that the cancer induces along the coronal third of the implant; the clinical and radiological appearance of such a histological finding is indistinguishable from peri-implantitis. (**B**): The deep infiltration of cancerous squamous cells progresses along the spires of the implants and invades the peri-implant bone (10× magnification). (**C**): This 40× magnification detail of the green area of picture B focuses on the linear deep peri-implant bone invasion, which simulates junctional epithelium.

**Figure 4:**
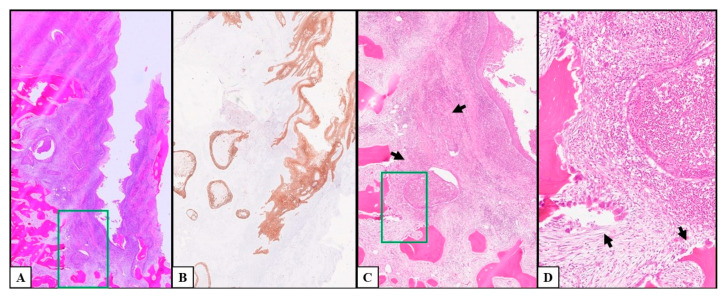
Histological pictures of a late lesion with immunohistochemical staining and increasing magnification. The figure shows the same late peri-implant squamous cell carcinoma at increasing magnifications (green rectangles). (**A**): Low-magnification (10×) picture showing bone resorption caused by the invasive squamous cell carcinoma. (**B**): Immunohistochemical staining for cytokeratins of a peri-implant squamous cell cancer infiltrating peri-implant bone and allowing the differential diagnosis from peri-implantitis (10× magnification). (**C**): Image showing 20× magnification details of picture (**A**); picture (**B**) shows the high density of lymphocytes and monocytes in the pseudo-follicular inflammatory infiltrate associated with the cancerous cell nests (arrows). (**D**): This 25× magnification picture focuses on the osteoclast-rich Howship’s lacunae of bone resorption.

The histological analysis of the surgical samples showed stage-linked neoplastic infiltration within the peri-implant gingival sulcus and spaces with different aspects due to the progression of the disease. After the excision of the lesions, en bloc bone resection, and histological examination of the depth of invasion (DOI), the reassessment of the pTNM showed 5 stage I cases (20.8%), 3 stage II cases (12.5%), and 2 stage III cases (8.3%); the remaining 14 cases showed pathological T4a cancers due to the presence of peri-implant bone invasion (58.4%) and underwent lymph node dissection, which allowed for further stadiation between stage IVa (8 patients; 33.4%) and IVb (6 patients; 25.0%). During the follow-up period, lymph nodal metastases occurred in two of the patients showing stage II peri-implant OSCCs (28.57%) and were reassessed as stage IVa; therefore, the total number of stage II tumors was 1 (4.2%), and the number of stage IVa tumors was 10 (41.7%). The early lesions (pT1–pT2) showed the superficial colonization of the juxta-implant superficial region of the epithelial lining of the sulcus with intraepithelial linear spreading, enlargement of the peri-implant spaces, and the initial resorption of superficial bone with a pocket-like appearance. Both the sulcus and chorion presented abundant phlogistic cells, such as lymphocytes, monocytes, and polymorphonucleates (PMNs) with intense vascular capillary proliferation. Lymph nodal metastases occurred in two of the patients showing early peri-implant OSCCs (25.0%). The late lesions (pT3–pT4a; Figure 3 and Figure 4) showed severe invasion of neoplastic epithelial cells and enlargement of peri-implant spaces associated with progressive resorption and infiltration of the bone, as well as implant lateral mobility; those tumors had higher vascularization with increasing vascular diameters and massive phlogistic infiltrate compared to the early stages; pT4 tumors showed bone invasion. Ten of the patients carrying late-stage peri-implant OSCCs developed lymph nodal metastases (62.5%).

The peri-implant OSCCs had large islands of neoplastic cells as the main pattern of infiltration in well-differentiated carcinomas (G1–G2) and single-cell infiltration in poorly differentiated carcinomas (G3). Ten tumors were well-differentiated (41.7%), eight were moderately differentiated (33.3%), and six were poorly differentiated (25.0%); in particular, the authors found that the histological architecture of moderately differentiated peri-implant OSCCs showed an overall well-differentiated epithelium except for the deepest part, where moderate differentiation occurred; the authors reported the same histological pattern also in poorly differentiated lesions. The DOI has proven to be more reliable than the superficial diameter of the tumor in determining the prognosis of patients with peri-implant OSCCs; anyway, the bone invasion and grading had a pivotal prognostic role. The peri-implant OSCCs that occurred in patients with previous histological diagnosis of PVL, OLP, and lichenoid dysplasia were G3. In patients who had previous oral cancers, there was high variability in the results regarding the grade of the tumors and the type of invasion. In the authors’ experience, a peri-implant OSCC may occupy the peri-implant sulcus without invading the crestal bone; the peri-implant bone resorption may occur because of the presence of concomitant PI as well as due to the inflammatory infiltration of the cancer. Anyway, the occurrence of peri-implant inflammation should be considered as a potential prognostic factor for the distance spread of the tumor. In the current study, 22 OSCCs showed concomitant PI-like inflammation (91.6%). The mean follow-up of the patients was 3.4 ± 1.0 years, whereas the mean DFS was 30.1 ± 17.9 months. The peri-implant OSCCs in patients with OPMD tumors showed a higher invasion index and worse prognosis than the tumors that occurred in those without OPMDs (mean DFS of 15.5 ± 7.7 months and 44.7 ± 12.1 months, respectively). In addition, a patient died because of the peri-implant OSCC in the group of those with previous OPMDs. In patients who had previous oral cancers, there was high variability in the prognoses.

## 4. Discussion

Table 3 summarizes the recommendations for early diagnosis, treatment, and follow-up that the authors derived from the study, which are discussed in the following subsections of the manuscript.

### 4.1. Peri-Implant Oral Squamous Cell Carcinoma and Peri-Implantitis: The Diagnostic Challenge

The first issue related to peri-implant OSCCs is the lack of a univocal nomenclature in literature; during the last few years, several authors have published on “malignancies,” thus involving OSCCs, metastases of distant tumors, and other kinds of cancers that occurred in the “vicinity of”, in “association with”, “adjacent to”, or “surrounding” dental implants [8]. The incidence of peri-implant OSCCs is higher in the mandible than in the maxilla and in women than in men, and the male–female ratio ranges between 1.5 and 2.5; the highest incidence occurs in the seventh decade of life [3,4,15,18]; the results of the current study align with those in the literature. The current study showed that peri-implant OSCCs occurred as single or multiple lesions which simulated PI both clinically and radiologically in most cases; PI affects 20% of implants and is chronic inflammatory resorption of the bone and soft tissues around the implants [1,4,10,12]. The clinical signs of PI include swelling, erythema, hypertrophy, suppuration, pocket formation, bone loss, ulcers, and even granular hyperplastic changes of the soft tissues; therefore, a differential diagnosis with malignant lesions is necessary in those cases to avoid severe diagnostic delays [1,3,4,12,15,16,24]. Other types of chronic peri-implant diseases have a prevalence between 14% and 54%; sometimes these lesions resemble tumors, thus requiring biopsy and histological examination to succeed in differential diagnosis [15,18]. However, surgery is the choice for PI after the failure of non-surgical treatment, which involves debridement, improvement of oral hygiene, antiseptics, antibiotics, and modifications to the implant surfaces [4]; PI responds to treatments and oral hygiene, whereas peri-implant OSCC progresses and infiltrates the surrounding soft and hard tissues [24]. In addition, several authors have raised the issue of surgeons who do not send peri-implant specimens for histological examination because they do not consider such biopsies clinically relevant [4,15,18]. The clinical–pathological findings of the current study suggest performing the histological examination of peri-implant specimens sampled during the surgical treatment of peri-implantitis; such findings agree with those of other authors [4,15,18]. In the literature, the rate of OSCC misdiagnosed as PI is variable and reaches 40.4%; several authors suggest that a cause of such a diagnostic difficulty is the lack of awareness of the malignancies mimicking PI among general dental practitioners [15,18]. According to the review by Kaplan et al., peri-implant OSCC suffers from a diagnostic delay of several months; in worst cases, such a delay may be over a year [15]. Dentists should record a detailed medical history of aerodigestive malignancies, OPMDs, and risk factors for OSCC in patients with PI; in addition, dentists should indicate a biopsy to achieve a definitive histological diagnosis in cases of resistant PI [1,16,24]. In addition, peri-implant OSCC has exophytic clinical variants too [1]; in the current study, 41.7% of the cancers occurred as exophytic mixed red and white lesions defined as micronodular leukoplakia-like presentations. Chainani-Wu et al. reported an OSCC that arose within an alveolar defect persisting one year after the removal of a failed implant in a patient with a history of oral cancer; the mucosa covering the defect did not show lesions suggesting the presence of a malignancy [10]. After implant placement, periodic oral and radiographic examinations are mandatory, especially in patients carrying known risk factors for OSCC, because peri-implant pathologies may involve malignancies [10,16]. Seriated radiographs allow the detection of persistent or growing bone defects that may need second-level radiologic exams and biopsy, even in cases of overlying healthy mucosa, such as during the early stages of OSCC [10]. Anyway, the current study’s findings showed that the radiological diagnosis of peri-implant OSCC was complicated because intraoral radiograms, panoramic radiograms, CT, and RMI had limitations that prevented the satisfactory visualization of the tumors using only one imaging method. Titanium scattering impaired the assessment of peri-implant bone by using panoramic radiograms and CBCTs; therefore, the authors suggest assessing the bone by using scattering-free imaging, such as intraoral periapical radiograms for a first-level radiologic examination and MRI for second-level diagnostic imaging, and recommend PET-CT in patients with late-stage tumors. The time between the placement of a dental implant and the development of a peri-implant malignancy is variable and ranges from several months to decades; the lag between the first primary OSCC and the peri-implant secondary tumor reaches up to several years [15]. In the current study, the peri-implant OSCCs developed after a mean period of 35.5 ± 13.8 months from the dental implant placement; the minimum was 16 months, and in this case the OSCC occurred in a patient with a previous OSCC. Seo et al. found that 75% of peri-implant OSCCs were stage IV; anyway, 80% of the OSCCs were well-differentiated [8]. Watanabe et al. reported the case of a maxillary stage IVb peri-implant OSCC, which occurred as an indurated ulcer associated with PI, severe mobility of subperiosteal dental implants, exposition of the metal framework, and palatal fistula in a 74-year-old male patient [12]. The results of the current study showed that 66.7% of the peri-implant OSCCs were of pathological stage IV, in line with the literature.

### 4.2. Risk Factors for Peri-Implant Oral Squamous Cell Carcinoma

Peri-implant malignancies account for up to 1.5% of oral cancers; in particular, some authors state that peri-implant OSCC is an infrequent malignancy, while other authors suggest that data regarding the incidence of peri-implant OSCC suffer from an under-reporting bias, whereas other authors have highlighted the increase in the number of case reports on peri-implant OSCC [1,2,15,24,25]. Sotorra-Figuerola et al. studied 111 soft tissue biopsies of peri-implant diseases and found OSCC in 3.6% of cases [18]. Other authors calculated a 3% incidence in patients with previous OSCC and 0.056% in those showing a medical history without OSCC [15]. In addition, the literature counts a few cases of peri-implant OSCC as the primary carcinoma because dental implants are the best choice for rehabilitation in patients with previous oral cancer; therefore, the incidence of peri-implant OSCC is high in those patients, and several authors believe that such an incidence will grow in the future [1,3,15,16]. The review of Ito et al. found that 65% of patients with peri-implant OSCC had a history of oral cancer excision and reconstruction involving dental implants [24]; therefore, such patients carried a risk of recurrence and metachronous OSCC independent of the dental implants. The review by Jané-Salas et al. gathered 19 cases of peri-implant OSCC; 10 had a previous history of OSCC, OPMDs, or cancer in other areas [1]; such trends align with other studies in the literature [10,15]. The results of the current study suggest that medical history provides information about the individual risk of developing peri-implant OSCCs; indeed, 14.3% of patients had already had another OSCC, and 38.1% carried an OPMD as a risk factor. Such results suggest that peri-implant OSCC occurs more frequently in patients with high individual susceptibility to developing oral cancers rather than in patients with conventional risk factors (such as a smoking habit alone) or those without risk factors. Peri-implant OSCC correlates with the conventional risk factors for oral cancer, such as heavy tobacco and alcohol consumption, and OPMDs such as leukoplakia, erythroplakia, OLP, and PVL [4,15,18]. Kaplan et al. described a patient with PVL who underwent surgery and radiotherapy because of two primary OSCCs and a peri-implant OSCC; these malignancies occurred in different sites after diagnosis of PVL within six years [15]. Raiser et al. found that 45.3% of peri-implant OSCCs occurred in patients with risk factors for oral cancer or OPMDs [4]. Ito et al. found that 7% of patients showing peri-implant OSCC had a history of OLP, which is an OPMD with a low-to-high risk of malignant transformation [24,26]. OLP is a chronic mucocutaneous autoimmune disease that occurs as episodic flare-ups [11]. OLP affects between 0.2% and 1.9% of the population; such disease affects patients between 30 and 70 years and women more than men [11]. The treatment of OLP involves cycles of local and systemic administration of immunosuppressive agents, such as corticosteroids locally or cyclosporine, azathioprine, or retinoids; thus, those patients develop a state of immunosuppression that increases the risk of cancers [11]. The malignant transformation of OLP occurs in up to 5.6% of cases during an observation period between six months and twenty years; therefore, OLP patients need a long-lasting follow-up [11]. The OLP patient described by Marini et al. developed a peri-implant OSCC in an area that was not previously affected by other lesions and during treatment with prednisone [11]. The authors suggested that such a malignancy occurred because of the contributions of the chronic inflammatory condition, the presence of the titanium implant, and the prolonged use of immunosuppressive drugs [11]. In the current study, 14.3% of patients had PVL, 19.0% had OLP, and 4.8% had lichenoid dysplasia. Another issue related to the literature on peri-implant OSCC is that several studies lack a multidisciplinary analysis involving oral surgeons, oncologists, maxillofacial surgeons, pathologists, and dentists; indeed, the first observational study that analyzed even the type of prosthesis and the constituting material as potential risk factors for peri-implant OSCC was published by Seo et al. in 2024 [8]. Those authors showed 20 peri-implant OSCCs that mainly occurred near porcelain fused to metal or gold partial fixed prostheses (cemented more than screwed); only two cases occurred in patients with total prostheses (cemented and removable, respectively) [8]. The results of the current study are in line with those data showing more partial prostheses than total; anyway, the percentage of those who wore overdentures was higher than the one reported by Seo et al. [8]. Both studies provide insufficient evidence to hypothesize a correlation between the type of prosthetic rehabilitation and peri-implant OSCC.

### 4.3. Oral Carcinogenesis and Dental Implants

The relationship between dental implants and carcinogenesis is unclear, and the current literature provides insufficient data to support any hypotheses [1,10,16,24,27]. Ito et al. excluded the hypothesis of dental implants as a risk factor for oral malignancies based on the literature and the lack of an increase in implant-related malignancies in the last 30 years [24]. Some authors suggested the generation of galvanic effects between the peri-implant mucosa and the rest of the oral mucosa, but epidemiological studies did not find a correlation between corrosive events and carcinogenesis in humans [3]. Other authors hypothesized the release of metallic ions and inflammatory cytokines as a risk factor around failing implants as promotors of peri-implant carcinogenesis [10,11,15]. Other authors suggest that the chronic inflammation within the gingival attachment to implants is a concurring factor for carcinogenesis [1,2,3,11,12,15]. Such inflammation arises from improperly fitted prostheses or poor oral hygiene, which is further responsible for PI [1,12]. However, the literature suggests several mechanisms for carcinogenesis associated with chronic inflammation, such as the excretion of cytokines and growth factors promoting neo-angiogenesis and the survival of tumoral cells and preventing their apoptosis [2,3]. Oral carcinogenesis occurs because of progressive genetic mutations in the oral mucosa; epithelial dysplasia, oral intraepithelial neoplasia (OIN), and carcinoma in situ (CIS) are early manifestations of oral carcinogenesis [2]. Noguchi et al. described a peri-implant OIN that occurred in a woman without risk factors for OSCC; the tumor showed immunohistochemical positivity for keratin-17, p53, p63, and Ki-67 in the basal layer. In addition, keratin 13 and p16 staining were negative [2]. Another pivotal factor in the progression of tumors is immune evasion, and OSCC evades immune response by expressing the Programmed Cell Death Ligand 1 (PDL-1) [28,29,30]. PDL-1 is a transmembrane protein that binds peri-tumoral and intra-tumoral CD8+ T lymphocytes; as a result, PDL-1 inactivates those cells and prevents the production of pro-inflammatory cytokines [28,29,30]. In line with the literature, the results of the current study highlighted the weight of the medical history and risk factors related to the health of the oral mucosa rather than finding dental implant-related risk factors.

### 4.4. Histological Examination of Peri-Implant Oral Squamous Cell Carcinoma

According to some hypotheses, the lack of periodontal ligaments associated with dental implants allows easy invasion from the epithelium to the cancellous bone by using the implant–bone interface as a route [1,3,11]. Anyway, histological analysis showed stage-linked neoplastic infiltration within the peri-implant gingival sulcus and spaces with different aspects due to the progression of the disease. Those results agree with the data reported by Verstraeten et al., who showed seven peri-implant OSCCs that did not show downward growth along dental implants and two invading the bone–implant junction [16]. Many studies showed that the cellular events occurring in soft tissues near the peri-implant OSCC induced the resorption of the bone rather than the malignancies themselves [16]. In line with the literature, the authors found inflammatory infiltrate showing stage-linked growth in all peri-implant OSCC specimens. Currently, pathologists cannot accurately distinguish all the types of inflammatory infiltrates in histological examination of peri-implant OSCC. For example, PI is a chronic nonspecific inflammation occurring within peri-implant soft tissues and is responsible for bone resorption; the typical cellular composition involves mainly plasma cells, polymorphonucleates, and a few lymphocytes [18,24,26,28,29]. Other chronic specific inflammations, such as autoimmune diseases, replicate cytological and histological features of PI and may occur in the peri-implant soft tissues; therefore, several authors define such cases as PI-like inflammation [18,24,26,28,29]. Eventually, even OSCC is a target of the immune response, especially of CD8+ lymphocytes and plasma cells, which show both peri-tumoral and intra-tumoral distributions. PI-related and OSCC-related inflammation contribute to bone resorption with a potential synergistic effect; thus, the authors suggest that the histological finding of both inflammatory infiltrates should be treated as a risk factor for bone infiltration. Future studies should establish the relationship between the amount of inflammatory infiltration and bone infiltration. Eventually, another diagnostic challenge of peri-implant OSCC is peri-implant nonspecific inflammatory hyperplasia (PINIH). In PINIH, the mucous epithelium can proliferate in response to the stimulation provided by the nonspecific chronic inflammation that involves lymphocytes, plasma cells, and fibroblasts [18]. The occurrence rate of PINIH in soft tissue biopsies of peri-implant diseases reaches 60.3% [18]. Few studies have addressed the importance of adapting the staging system and the type of resection based on the distance of OSCC from dental implants because those prosthetics could facilitate tumor invasion in soft tissues and peri-implant bone [16]. Verstraeten et al. stated that a peri-implant OSCC should not have dedicated staging and treatment planning because of the lack of evidence that the bone–implant junction is a route for tumor invasion [16]. Based on their experience, the authors suggest that the main histological prognostic parameters for peri-implant OSCCs are the DOI, grade, and bone invasion. In addition, the surgical excision of peri-implant OSCC requires a demolitive rather than conservative approach. Therefore, planning the surgical excision and the following prosthetic rehabilitation is complex.

### 4.5. Limitations and Implications for Future Research

The current study suffers from several limitations, such as the observational retrospective design, the data having been obtained from a single center, the small sample size, and the limited generalizability. Future studies should focus on a prospective design by gathering data from several centers to increase the sample size and strengthen the generalizability of the findings.

## 5. Conclusions

The results of this study showed that peri-implant OSCCs occurred twice as often in patients who had already had oral cancers and OPMDs compared to those without such risk factors. In addition, the peri-implant OSCCs required demolitive surgery rather than conservative excision, and the prognosis of patients strictly depended on the grade of the cancer. In the authors’ experience, the diagnosis of peri-implant OSCCs was complicated because of the PI-like clinical presentation and the limitations in imaging, which prevented the satisfactory visualization of those cancers using only one method; however, most patients included in the study received a diagnosis of early-stage peri-implant OSCC.

## Figures and Tables

**Table 1:** Summary of clinical and pathological findings.

Outcomes	Results					
Sample Size (*n*)	21					
Female/Male (*n*/*n*; Ratio)	13/8; 1.6					
Age (Mean ± SD)	70.6 ± 11.7 years					
Number of OSCCs	24					
Number of Implants Involved	50					
Clinical Presentation (*n*; %)	Erythroplakia-like	Exophytic mixed				
	14; 58.3%	10; 41.7%				
cTNM Staging (*n*; %)	Stage I	Stage II	Stage III	Stage IVa	Stage IVb	Stage IVc
	9; 37.5%	12; 50.0%	1; 4.2%	2; 8.3%	0; 0.0%	0; 0.0%
pTNM Staging (*n*; %)	Stage I	Stage II	Stage III	Stage IVa	Stage IVb	Stage IVc
	5; 20.8%	1; 4.2%	2; 8.3%	10; 41.7%	6; 25.0%	0; 0.0%
Grading (*n*; %)	G1	G2	G3			
	10; 41.7%	8; 33.3%	6; 25.0%			
Presence of PI-Like Inflammation (*n*; %)	22; 91.6%					
Follow-Up (Mean ± SD)	3.4 ± 1.0 years					
Disease-Free Survival (Mean ± SD)	30.1 ± 17.9 months					

**Table 2:** Summary of findings on the risk factors for peri-implant OSCCs.

Medical History	*n*	%
Previous oral squamous cell carcinoma (OSCC) and former smokers	3	14.3%
Proliferative verrucous leukoplakia (PVL)	4	19.0%
Oral lichen planus (OLP)	3	14.3%
Oral lichen planus (OLP) AND smoking habit	2	9.5%
Lichenoid dysplasia	1	4.8%
Lichenoid dysplasia AND smoking habit	1	4.8%
Smoking habit	3	14.3%
Negative medical history for risk factors	4	19.0%

**Table 3:** Summary of recommendations for early diagnosis, efficient treatment, and follow-up of peri-implant oral squamous cell carcinoma.

PHASE	RECOMMENDATIONS
Medical History	Check for previous oral cancers, oral potentially malignant disorders, and smoking habits.
Clinical Examination	Use vital stainings (toluidine blue and Lugol’s solution) to study erythroplakia-like and exophytic mixed lesions of peri-implant mucosa.
	Consider as suspicious all lesions persisting after two weeks from the decontamination of peri-implant pockets.
	Check for multiple synchronous suspicious lesions.
	For an optimal clinical examination, study the suspicious lesions after removing the dental prosthesis.
Imaging	Use scattering-free methods, such as intraoral radiograms and magnetic resonance imaging, to study the bone associated with suspicious peri-implant lesions.
Diagnostic Interventions	Gather mucosal samples for histological examination in case of surgery for treatment of peri-implantitis.
	Perform biopsy and histological examination of suspicious peri-implant lesions; the most experienced operators can perform peri-implant sulcus sampling for cytologic examination.
Surgical Excision	The demolitive approach is more recommendable than the conservative.
Follow-Up	The worse the histological grading of cancer, the stricter the follow-up should be.

## Data Availability

The raw data supporting the conclusions of this article will be made available by the authors on request.
